# Chagas Disease Drug Discovery in Latin America—A Mini Review of Antiparasitic Agents Explored Between 2010 and 2021

**DOI:** 10.3389/fchem.2021.771143

**Published:** 2021-10-28

**Authors:** Ramon G. de Oliveira, Luiza R. Cruz, María C. Mollo, Luiz C. Dias, Jadel M. Kratz

**Affiliations:** ^1^ Laboratory of Synthetic Organic Chemistry, Institute of Chemistry, University of Campinas (UNICAMP), Campinas, Brazil; ^2^ Drugs for Neglected Diseases Initiative (DND*i*) Latin America, Rio de Janeiro, Brazil

**Keywords:** *Trypanosoma cruzi*, screening, hit identification, hit to lead, lead optimization, medicinal chemistry, neglected tropical diseases

## Abstract

Chagas disease is a neglected tropical disease caused by the protozoan parasite *Trypanosoma cruzi* that endangers almost 70 million people worldwide. The only two drugs that are currently approved for its treatment, benznidazole and nifurtimox, have controversial efficacy in adults and restricting safety issues, leaving thousands of patients without a suitable treatment. The neglect of Chagas disease is further illustrated by the lack of a robust and diverse drug discovery and development portfolio of new chemical entities, and it is of paramount importance to build a strong research and development network for antichagasic drugs. Focusing on drug discovery programs led by scientists based in Latin America, the main endemic region for this disease, we discuss herein what has been published in the last decade in terms of identification of new antiparasitic drugs to treat Chagas disease, shining a spotlight on the origin, chemical diversity, level of characterization of hits, and strategies used for optimization of lead compounds. Finally, we identify strengths and weaknesses in these drug discovery campaigns and highlight the importance of multidisciplinary collaboration and knowledge sharing.

## Introduction

Chagas disease (CD) is a neglected tropical disease (NTD) caused by the protozoan parasite *Trypanosoma cruzi* (*T. cruzi*). The disease affects around 6 million people worldwide, with over 30,000 new cases and 12,000 deaths per year (Pan-American Health Organization (PAHO), 2021). After the initial infection, the majority of cases progress to an asymptomatic chronic phase, which can evolve into the chronic determinate form of the disease years later, with around 30% of patients presenting irreversible damage to the heart and nervous and digestive systems (WHO, 2021).

The current antiparasitic treatment is restricted to two drugs, benznidazole (BZN) and nifurtimox (NFX). The treatment is highly effective if given shortly after infection, at the onset of the acute phase, and for cases of congenital transmission or reactivation. But these treatments have substantial limitations, particularly for chronic indeterminate patients, including long treatment duration and tolerability issues (Junior et al., 2017).

**FIGURE 1:**
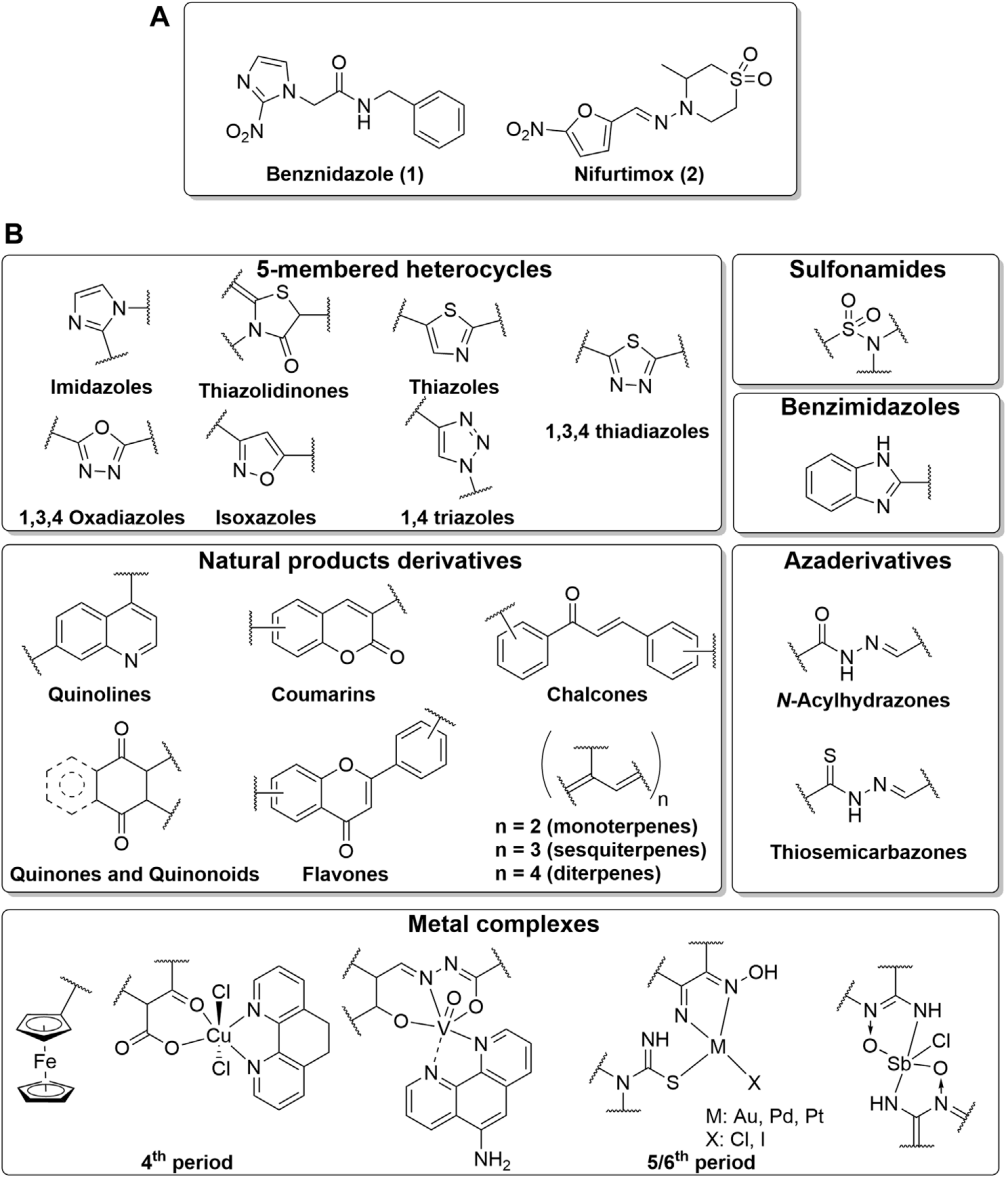
Representative chemical scaffolds. **(A)** Current drugs used for treatment of Chagas disease, benznidazole (1) and nifurtimox (2). **(B)** Most common chemical scaffolds reported in the evaluated publications.

Discovered in the 1970s, BZN (1, Figure 1A) is a 2-nitroimidazole pro-drug activated through the reduction of the nitro group by parasite nitroreductases, a process that generates reactive metabolites that ultimately disrupt parasite cell machinery and are responsible for the trypanocidal effect (Kratz et al., 2018). This nitro reduction can generate reactive oxygen species in humans, which are associated with the main toxicological findings, including carcinogenicity, teratogenicity, and genotoxicity (similar to other nitroimidazole-containing drugs). In the clinic, 20–25% of patients present dermatitis from hypersensitivity to the drug and this, together with digestive intolerance, is the main reason for treatment interruption (Gaspar et al., 2015). Similarly, NFX (**2**, Figure 1A), a 5-nitrofuran derivative, is also activated via parasite nitro reduction producing reactive metabolites. NFX has an even higher frequency of adverse effects (Gaspar et al., 2015).

Regrettably, CD is a neglected disease, with modest investment and limited coordination of research and development (R&D), resulting in knowledge gaps that prevent scientific advancement. Very few candidates have been progressed from the early stages of drug discovery (DD) to clinical settings, with recent clinical studies mainly focusing on regimen improvement of approved drugs, such as the recently published BENDITA trial (Torrico et al., 2021). The few new chemical entities (NCEs) that have been clinically evaluated were unfortunately not successful (e.g., fexinidazole, posaconazole and a ravuconazole prodrug–the last two targeting the ergosterol synthesis pathway) (Molina et al., 2014; Morillo et al., 2015; Torrico et al., 2018; Drugs for Neglected Diseases initiative (DND*i*), 2020).

Given these limitations and the unmet medical needs for Chagas patients, development of new, safer, and affordable treatments is imperative and should be at the frontline of DD programs around the world, particularly in endemic areas. This mini review brings an overview of recent early-stage projects in Latin America that are focused on the identification antichagasic drugs. It highlights some of the key aspects of these DD efforts and discusses the challenges and opportunities for the CD research community.

## Research Methodology

The scope of this mini review is limited to manuscripts published by Latin American groups reporting the investigation of small molecules/NCEs and plant extracts with antiparasitic activity against *T. cruzi*. Manuscripts solely reporting investigations of parasite biology, diagnostics, vaccines, biologicals, and new formulations of known drugs were not considered.

A literature search was conducted using the Web of Science database using “*Trypanosoma cruzi*” as a single keyword for the primary search between 01/01/2010 and 31/03/2021. The initial search returned 8,683 entries. The filters “chemistry medicinal” and “chemistry organic” were applied, reducing the total to 1,029 entries. A second filter was applied restricting the “document type” to “articles and early access” (857 entries), followed by another that restricted documents to those with an author affiliated to a Latin American institution; this resulted in 543 entries. This subset of manuscripts was carefully inspected, and those whose first and/or corresponding authors did not meet the affiliation criteria were excluded. This final set of 343 published articles was analyzed in depth for this mini review.

To extract unique compound information and analysis of physiochemical properties, document object identifiers (DOI) were cross searched in the ChEMBL database (release 29) (Gaulton et al., 2017). Entries with no synthesized compounds and/or without isolated compounds (only complex mixtures from plant extracts) were excluded (34/343). A ChEMBL database search returned 136 entries, representing approximately 45% of the articles. Compound names and SMILES were extracted with associated biological data, imported into StarDrop® (Optibrium Ltd.) and manually inspected. Positive/negative controls and other undesired molecules (cofactors, duplicates) were excluded. The final set comprising 2,846 unique compounds from 136 different articles was used in this mini review.

## Hit Identification and Initial Chemical Expansion

The variety of approaches that are available for initial screening and hit identification is enormous. Strategies such as high-throughput screening (HTS) of large compound libraries, combinatorial chemistry, and prospection of natural products (NP) rely on a serendipity approach. Whereas methods such as structure-based drug design (SBDD) and ligand-based drug design (LBDD), both frequently supported by virtual screening (VS), and design backed by literature- and patent-derived information represent knowledge-based approaches (Bleicher et al., 2003; Hughes et al., 2011).

Almost a third of the published articles reported hits originating from natural products: compounds extracted from plants and other organisms or inspired by naturally occurring compounds. This is not surprising considering the huge ethnobiology and ethnopharmacology potential of the region and the traditional R&D activities of Latin American academic groups (Calixto, 2005). Natural product-based drugs are one of the cornerstones of anti-infectious therapy, but the discovery of new drugs from natural sources faces specific challenges, such as isolation and characterization of pure compounds and balancing drug-like properties; however, this field has been resurging in recent years, mainly due to advances in automation and modern omics techniques (Harvey et al., 2015; Calixto, 2019; Atanasov et al., 2021).

The remaining articles report different hit identification methods: approved drugs analogues, VS, fragment libraries, HTS or medium-throughput screening of compound libraries. Most articles, however, report compounds originating from previous research knowledge obtained within the same research group or from the literature. These latter reports usually describe the design (employing a mix of classical medicinal chemistry methods, such as hybridization, bioisosterism, and privileged structures), synthesis, and evaluation of a small expansion of the previously published dataset.

Computational methods are now an intrinsic part of modern DD (Ferreira et al., 2019). Tools such as SBDD, LBDD, VS and many others are widely used in academia and industry. In this review, 84/343 papers (<25%) presented molecular docking data for the compounds (i.e., conformation prediction, binding mode, and molecular interactions), but only four actively used this structural and molecular recognition information to aide in the design and optimization of compounds.

Interestingly, two articles reported compounds originating from freely available boxes of annotated and drug-like compounds: one from the Open-Source Chagas Box (Peña et al., 2015) and the other from the Malaria Box (Voorhis et al., 2016). Open resource tools are available to catalyze research in NTDs DD and represent a way to overcome some of the challenges for academic projects, such as access to large compound libraries.

A summary of the most common chemical scaffolds is depicted in Figure 1B. Indeed, most of the scaffolds are frequently present in natural products: quinones and quinonoids, quinolines, chalcones, terpenes, and flavones make up almost 30% of the reported scaffolds. Additionally, thiosemicarbazones–known cysteine proteases inhibitors (Linciano et al., 2018)—and hydrazones were also described in around 10% of the publications.

## Quality of Hits

The quality of the chemical starting points is a key factor to be considered since poor-quality hits have a greater likelihood of failure during the downstream hit-to-lead (H2L) and lead optimization (LO) phases. Using high standards and well-considered criteria for go/no-go decisions, and profiling hits against target candidate profiles (TCPs), will increase the chances of success and help focus R&D resources on chemical series that stand a reasonable chance of becoming a drug.

Drug likeness is a term used to describe the physicochemical space that is more likely to afford oral drugs (Mignani et al., 2018; Shultz, 2018). This concept is particularly useful during early DD stages. For instance, getting the right absorption, distribution, metabolism, and excretion (ADME) properties is crucial for clinical success since undesired pharmacokinetics (PK) and toxicity are the main reasons for clinical failure. There are published guidelines and free tools available to help researchers find the right drug-like space for their candidates (Ritchie et al., 2011; Daina et al., 2017; Jia et al., 2020; Leeson et al., 2021). However, only 17% of the articles reported any calculated physicochemical or ADME descriptors (mostly logP and other rule-of-five descriptors) and <5% presented any experimental ADME data.

**FIGURE 2:**
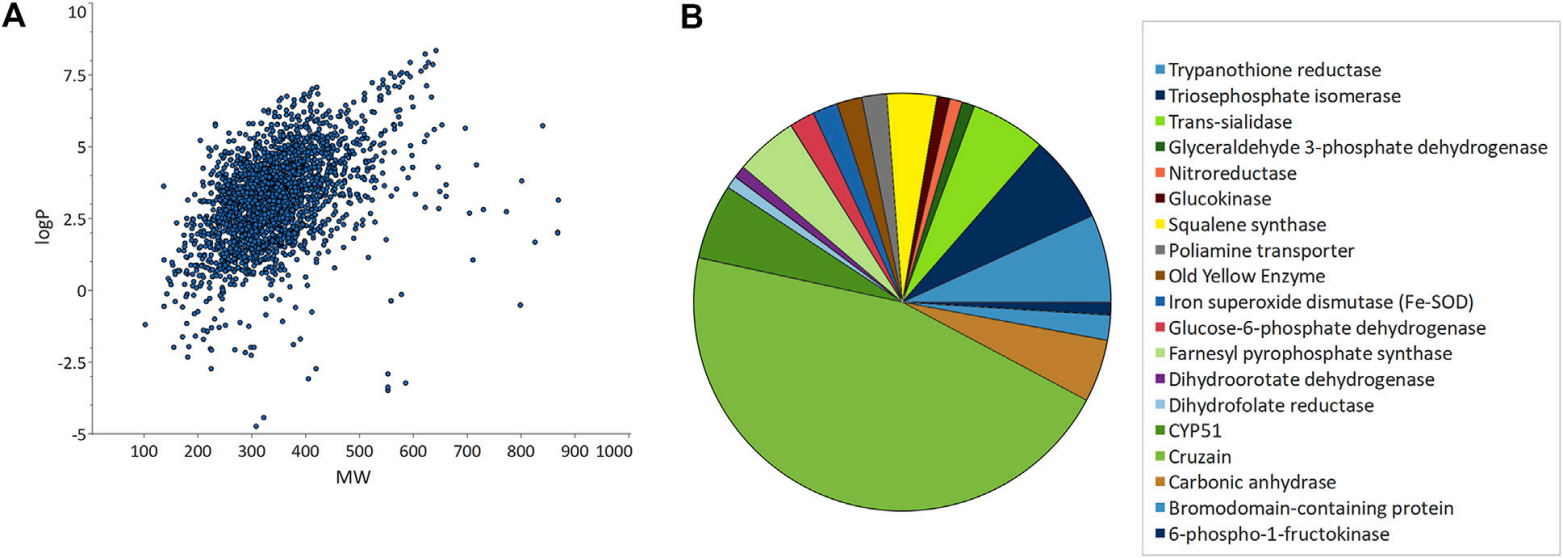
Unique compounds data obtained from ChEMBL. **(A)** Calculated lipophilicity (clogP) vs. molecular weight (MW) plot. **(B)** Molecular targets commonly explored in Chagas disease drug-discovery campaigns.

From the 2,846 unique chemical structures retrieved from the articles, it is possible to get an overview of the chemical landscape explored. A table showing calculated descriptors is available as Supplementary Material. Most compounds have a drug-like molecular weight (MW), ranging between 200 and 400 Da, with a few exceptions of homodimers, dendrimers, and natural products (Figure 2A). At initial stages of optimization projects, it is desirable that lipophilicity stays within a reasonable range to avoid unspecific and off-targets effects, and to allow further structural modifications. Approximately 93% of the structures had lipophilicity (clogP) between 2 and 5, which shows that most of the compounds are prone to scaffold exploration, structure-activity relationship (SAR) studies and show potential for multiparametric optimization.

Conversely, some functional groups/fragments are known for their intrinsic toxicity and/or assay interference. Applying a Pan-Assay Interference Compounds (PAINS) filter available in StarDrop (Hann et al., 1999), 15% of the compounds were flagged, raising concerns of false positives or a higher chance of failure during *in vivo* studies (Baell et al., 2013; Yang et al., 2021).

In terms of *in vitro* potency and selectivity, recently published TCPs can guide researchers progressing hits towards lead-stage compounds. Katsuno et al. (2015) and Chatelain and Ioset (2018) state that a promising Chagas hit should have a half-maximal inhibitory concentration (IC_50_) <5–10 µM against the intracellular amastigote form of *T. cruzi*, combined with at least 10-fold selectivity over the host cell line. As discussed in the next session, the heterogeneity of *in vitro* assays used across CD research makes comparison of the compounds published in different articles complex. Of the 343 research articles evaluated in this mini review, <20% had at least one compound (against any strain or parasite life stage) with IC_50_ < 1 μM, around 30% with IC_50_ between 1 and 5 μM, and almost 50% had all compounds with IC_50_ > 5 µM.

## Types of Drug Discovery Campaign

In general, there are two types of DD projects, phenotypic- and target-based campaigns, and is common to see both approaches used together (Gilbert, 2013). Phenotypic campaigns rely on phenotypic measures of response, e.g., an observable within a biological system, whereas target-based campaigns rely on a specific target that has an important role in the disease (Frearson et al., 2007; Osorio-Méndez and Cevallos, 2019).

Phenotypic-based approaches have been the standard starting point for CD DD programs, mainly due to the shortage of genetically and chemically validated targets. Almost 70% of the studies evaluated in this mini review report pure phenotypic campaigns (a molecular target was not used for hit identification nor was a target deconvolution effort pursued). Many types of phenotypic-based *in vitro* assays were used, with different throughput rates and readouts. Colorimetric-based assays were the most common assays, but there is clear heterogeneity in the *in vitro* assays conditions, with variable parasite strains and discrete type units (DTUs), parasite forms, controls, and mammalian host cell lines.

The three different *T. cruzi* life forms play different roles in parasitism and clinical symptoms. As such, it is suggested that intracellular amastigotes, prevalent in the chronic phase, should be prioritized when screening for *in vitro* efficacy, which generates both antiparasitic and cytotoxicity data. However, only 37% of the studies used *T. cruzi* intracellular amastigote assays, while the majority used arguably less disease-relevant parasite forms. Additionally, there are seven *T. cruzi* DTUs, with different sensitivity to approved drugs (Zingales, 2018). It is recommended that more advanced screening campaigns include a panel of DTUs in the screening cascade, however, less than 10% of the papers reported data against at least two different strains (Chatelain and Ioset, 2018; Kratz et al., 2021).

Despite the prevalence of phenotypic-based reports, there is a wide range of molecular targets being explored (Figure 2B). CYP51, the *T. cruzi* sterol 14-alpha demethylase, is probably the most well-known target in CD (Urbina, 2009; Rao et al., 2019). Surprisingly, only six articles reported the identification/pursue of CYP51 inhibitors, likely because of the recent failure of posaconazole in the clinic (Molina et al., 2014). Another interesting example of an extensively explored target is cruzain, which appeared in 47 reports (the most prevalent target). Cruzain is the most abundant *T. cruzi* cysteine protease and is critical for parasite survival. High-quality crystal structures are now widely available to support structure-based DD. Although there have been significant efforts to validate this target genetically and chemically for over 20 years, translational challenges still persist (i.e., from cell-free to whole-cell activity) (Barr et al., 2005; Martinez-Mayorga et al., 2015; Kratz et al., 2021).

Target deconvolution (identification of the molecular target of a phenotypic hit) is a powerful enabling tool to LO efforts, providing important information about the pharmacology and toxicology of a particular chemical series. However, the identification of a molecular target and/or mode of action is a complex, time- and resource-consuming process. Many orthogonal assays, as well as a broad range of expertise, are required (Wyatt et al., 2011; Field et al., 2017). In spite of the complexity of the process and inherent difficulties of genetically handling the parasite (Terstappen et al., 2007; Altamura et al., 2020), recent examples of successful target deconvolution efforts for CD include cytochrome *b* (Khare et al., 2015; Wall et al., 2020) and the proteasome (Khare et al., 2016). In this review, none of the publications reported the use of wide target deconvolution techniques, although some groups reported efforts to identify the mechanism of action of compounds using assays that monitor free radicals, mitochondria, or cell morphology.

## Progression of Hits and Lead Optimization

In order to progress to more advanced studies and achieve the preclinical/clinical candidate stage, early-stage compounds usually go through multiple cycles of multiparametric optimization. This process uses a structured screening cascade and progression decisions are based on clear cut-off criteria. These iterative cycles of design/synthesis/test/analysis usually yield hundreds (or even thousands) of compounds, and are necessary to overcome gaps in the SAR, as well as to balance the biological properties of chemical series, such as potency, selectivity, ADME (e.g., lipophilicity, cellular permeability, aqueous solubility, and hepatic clearance), and toxicity.

The vast majority of the publications evaluated in this mini review (∼95%) do not formally report rounds of data-driven optimization of compounds. It is fair to assume no formal LO program for any antitrypanosomal compound has been conducted in Latin America during the period covered by this review. The scope of the publications is mainly restricted to hit identification and limited chemical expansion, with approximately half of the studies reporting less than 10 different analogues per article.

Finally, only 25 publications (<10%) reported compounds that were progressed to *in vivo* studies. Of these, two studies were restricted to exploratory toxicology studies in murine models. Twenty-three studies reported the evaluation of compounds in the acute *T. cruzi* infection model in mice, some combined with toxicological endpoints, and two studies also reported the evaluation of compounds using the chronic model of infection in mice.

Although it is crucial to progress compounds to *in vivo* proof-of-concept studies to assess the antiparasitic potential of a candidate, it is fundamentally important to combine pharmacodynamic (PD) information with drug metabolism and pharmacokinetics (DMPK) and safety data (Chatelain and Konar, 2015; Katsuno et al., 2015; Martín-Escolano et al., 2020; Kratz et al., 2021). Only two studies generated experimental information about *in vitro* mouse microsomal clearance before progressing to animal models, and only two studies reported *in vivo* plasmatic exposure data in the same species used for the CD model. Without systematic ADME data and combined PKPD *in vivo* information it is virtually impossible to optimize the dose regimen *in vivo*, understand failures that are related to lack of systemic exposure, and/or establish exposure-response relationships for the chemical series.

## Perspectives

This mini review shows that during the last 10 years a significant number of published articles led by researchers affiliated to Latin American institutions reported efforts in CD DD. These are mainly academic projects, corroborating the importance of academia in the training of highly skilled researchers and the creation of knowledge and technologies. But despite the positive contributions of the region to the global CD R&D landscape, some limitations are evident. In this view, a few adjustments are discussed below with the goal of encouraging the exploration of the full scientific potential in Latin America and promoting the much-needed advancements in the field of medicinal chemistry and NTDs DD that, ultimately, might result in benefits to CD patients.

Developing NCEs is a major challenge and an impractical task for a single group or organization. Multidisciplinary international collaboration is key to modern DD and even more so in the CD field where long-lasting challenges are still present and there are obvious expertise gaps in the region (e.g., availability of ADME assays and integration of PK studies with disease models). In fact, about half of the articles evaluated in this review had coauthors from groups outside the corresponding author’s country, and only about a third had coauthors from outside Latin America.

Most articles described only primary screening campaigns that stopped at the hit identification stage, or presented only limited chemical expansion and SAR exploration, never reaching the level of a full LO program where key data is usually generated allowing the progression of candidates to the preclinical regulatory level and subsequently first-in-human studies. In fact, very few studies generated multiparametric data that was obtained through a structured screening cascade (e.g., combined *in vitro* potency, selectivity, and ADME/PK data) or guided project decisions based on a clear TCP. It will be virtually impossible to progress a NCE to the clinical setting if poor quality hits are selected as starting points and if projects remain restricted to the early H2L stages with limited information available.

Therefore, it is of utmost importance for the CD community in Latin America to fill in these historical technological gaps by coordinating its efforts towards data-sharing and organization of multidisciplinary collaborations, by implementing ADME platforms in the region, and by integrating DMPK into early DD projects to increase the chances of identifying a novel clinical candidate for CD.
